# Efficient Colorimetric Fluoride Anion Chemosensors With Varied Colors Based on Simple Aminobenzodifuranone Organic Π-Conjugated Dyes

**DOI:** 10.3389/fchem.2020.00231

**Published:** 2020-04-15

**Authors:** Zhi Feng Deng, Rui Li, Jie Ting Geng, Meng Zheng, Lei Quan Li, Xin Shi, Wen Qi Ren, Zi Yue Meng, Zhuo Ting Ji, Jing Hua

**Affiliations:** ^1^National and Local Joint Engineering Laboratory for Slag Comprehensive Utilization and Environmental Technology, School of Materials Science and Engineering, Shaanxi University of Technology (SNUT), Hanzhong, China; ^2^Key Laboratory of Rubber-Plastics of Ministry of Education, Shandong Province, School of Polymer Science & Engineering, Qingdao University of Science & Technology, Qingdao, China

**Keywords:** sensors, dyes, aminobenzodifuranone, fluoride anion, hydrogen bonding, detection

## Abstract

High selectivity and sensitivity detection of fluoride anions (F^−^) in an organic solution by the naked eye has always been a challenge. In this investigation, a simple compound based on aminobenzodifuranone (ABDF) was designed and synthesized. Deprotonation of the amino moiety caused by F^−^ is responsible for a color change from dark blue to various colors (colorless, yellow, orange, and red) in different common organic solvents due to a blue shift over 200 nm in the UV/Vis spectrum. The color change is quite visible to the naked eye under ambient light. The detection limit for F^−^ can reach a concentration of as low as 5.0 × 10^−7^ M with high selectivity, even in a solution containing multiple anions.

## Highlights

- A simple aminobenzodifuranone (ABDF) dye was synthesized and used as fluoride anion sensor.
- ABDF is highly selectivity and sensitivity for fluoride anion with detection limitation as low as 5 × 10^−7^ M.
- ABDF detect fluoride anion in different solvent in response varied colors which is sensitive for the naked eye detection.
- The deprotonation of amino moiety caused by F^−^ is responsible for the color change which is sensitive for the naked eye.

## Introduction

As one of the smallest anions and the most electronegative atom, fluoride with high charge density has special functions in chemical industry, organic synthesis, military fields, and medical and biological processes (Wade et al., 2010; Zhou et al., 2014). Environmental pollution by fluoride anions (F^−^) is one of the main problems to be addressed in the treatment of drinking water (Li et al., 2017). With the rapid development of the chemical industry, F^−^ has come to exist not only in the aqueous environment but also in organic media such as fluoride anion-containing pesticides and waste organic liquor (Clark, 1980). Recognition and detection of fluoride in organic solvent with a simple sensor and minimal instrumental assistance are imperative for practical applications (Yang et al., 2013; Kaur and Choi, 2015; Curnow et al., 2018; Tang et al., 2018; Murfin et al., 2020). Among various F^−^ sensors, fluorescence sensors have been widely investigated due to their multiple advantages (Li et al., 2017). However, those allowing naked-eye detection with high selectively and sensitively are more interesting, promising, and challenging due to offering simple and straightforward F^−^ detection without using auxiliary equipment.

**Figure 1:**
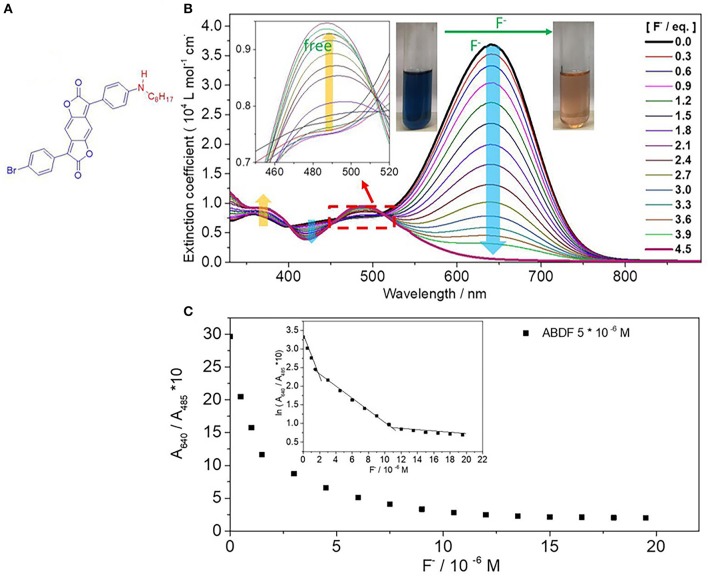
**(A)** Chemical structure of ABDF. **(B)** UV/Vis absorption spectra of ABDF (1.0 × 10^−5^ M) in the presence of F^−^ (0–4.5 eq.) in THF. The insets are the enlargement of UV/Vis spectra from 450 to 520 nm and color change of ABDF upon adding F^−^. **(C)** Ten times the absorption intensity ratio of ABDF (5 × 10^−6^ M after mixing with F^−^ in THF) between 640 and 485 nm (A_640_/A_485_ nm) vs. fluoride anion concentration. The inset is the natural logarithm of 10 times the absorption intensity ratio A_640_/A_485_ nm vs. fluoride anion concentration.

Fluoride anions exist in various organic solutions during chemical synthesis. Many studies have been reported on that focused on materials that detect F^−^ in a single organic solution, such as tetrahydrofuran (Sun et al., 2017), acetonitrile (Han et al., 2007), or chloroform (Lee et al., 2001). To the best of our knowledge, F^−^ sensors with naked-eye detection in different solutions, signaling via color changes, have not yet been developed. Such sensors might be possible in materials with solvatochromic behavior since they exhibit different colors in different solvents. Amino-substituted benzodifuranone (ABDF, Figure 1A) is a deep blue-colored dye that has attracted our attention due to its good photo-stability and interesting solvatochromic behavior (Zhang et al., 2014; Deng et al., 2018). The NH unit in the ABDF dye can undergo hydrogen bonding with the carbonyl group in the ABDF core (Deng et al., 2018). Zhang and others reported that the hydrogen bond formed could be associated with the molecular packing in the solid state (Yao et al., 2015; Oh et al., 2016). Further, Du and Hu's research group reported that thin film with good packing or crystallinity is beneficial for charge carrier mobility (Hu et al., 2000; Zhang et al., 2017, 2018; Du et al., 2018; Muhammad et al., 2020). Hence, the small ABDF molecule has been used in organic field-effect transistors with high performance, as reported by our group (Deng et al., 2018). Very recently, Zhang and co-authors also developed pigments with an amino unit in the color-changing coating and a UV-resistant coating (Zeng et al., 2018, 2019; Zhang et al., 2020). In this work, the amino units might be deprotonation in F^−^ solution, leading to a color variance detectable by the naked eye. ABDF exhibits diverse colors in different solutions, which may cause this chromophore to exhibit varied colors in different solvents. In addition, ABDF is soluble in most common organic solutions, with a high extinction coefficient of up to 6.5 × 10^4^ L mol^−1^ cm^−1^ (in dichloromethane, DCM) (Zhang et al., 2018). This could lead ABDF to be highly sensitive to F^−^. In this work, ABDF as a chemosensor for F^−^ was firstly investigated.

## Results and Discussion

The synthesis of ABDF was described in our previous work with B. Tieke' groups (Zhang et al., 2018; Muhammad et al., 2020), and the details are described in the Supporting Information. The interaction between ABDF chromophore and F^−^ was firstly investigated in THF solution through spectrophotometric titration experiments. A standard solution of tetrabutylammonium fluoride (TBAF, 1.0 × 10^−3^ M) was gradually added into a 1.0 × 10^−5^ M solution of ABDF in THF. As can be seen from Figure 1B, ABDF exhibits a high extinction coefficient of up to 3.7 × 10^4^ L mol^−1^ cm^−1^. With progressive addition of fluoride anion, the absorption intensity of the spectral peak at 640 nm is gradually decreased and finally disappears, while a new absorption peak at 485 nm emerges and increases. In addition, the absorption intensity at a broad band between 398 and 445 nm exhibits a steady decrease; meanwhile, the absorption intensity band between 330 and 400 nm is increased. The inset of Figure 1B shows that the color of the ABDF solution is changed from dark blue to orange upon adding F^−^. Both colors can be sensed by the naked eye under ambient light. ABDF is thus a F^−^ sensor allowing the naked-eye detection.

To examine the sensitivity of the sensors toward F^−^ in THF solution, the ratiometric curve was investigated. Figure 1C shows the ratiometric curves of the absorption intensity ratio of ABDF in THF between 640 and 485 nm vs. F^−^ concentration, which seems to be a quadratic function. The natural logarithm of Figure 1C results in the inset figure. As can be seen from the inset of Figure 1C, there are three slopes in the correlation, and the cross points of fitting lines are at 1.7 × 10^−6^ M and 1.1 × 10^−5^ M, respectively. The linear fit relationships between absorption intensity and F^−^ concentration are: ln(A_640_/A_485_ nm) = −0.57 [F^−^] + 3.31 (5.0 × 10^−7^ M ≤ [F^−^] < 1.7 × 10^−6^ M), ln(A_640_/A_485_ nm) = −0.16 [F^−^] + 2.60 (1.7 × 10^−6^ M ≤ [F^−^] < 1.1 × 10^−5^ M), and ln(A_640_/A_485_ nm) = −0.16 [F^−^] + 2.60 (1.1 × 10^−5^ M ≤ [F^−^] ≤ 2.0 × 10^−5^ M). The signal output discrimination is still distinct at a F^−^ concentration of 5.0 × 10^−7^ M, which indicates that the sensor is highly sensitive for F^−^ concentration and can offer quantitative information.

**Figure 2:**
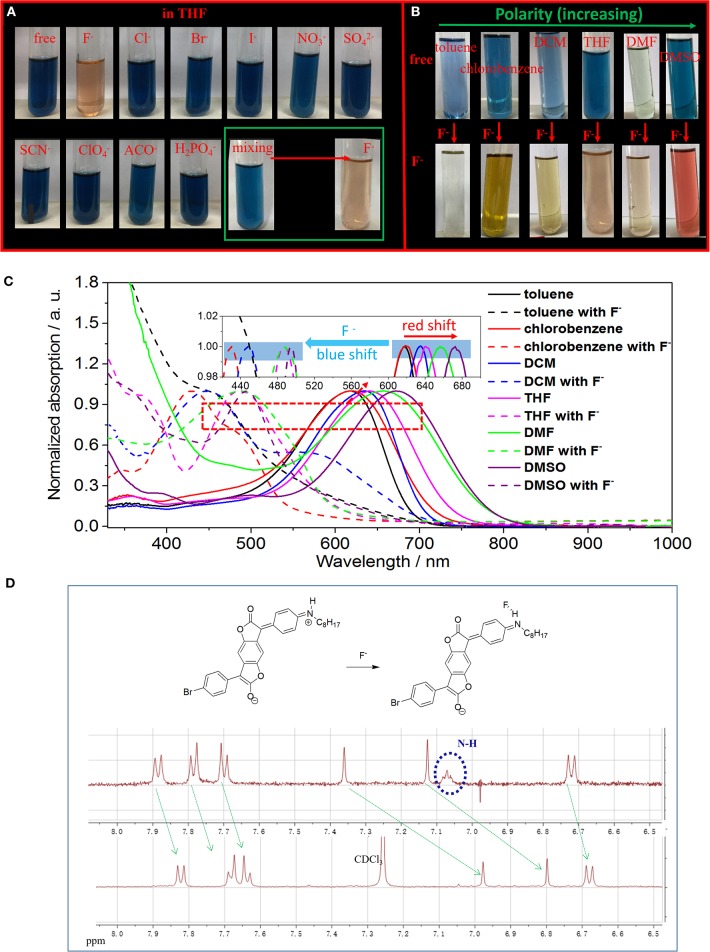
**(A)** Photographs of ABDF in THF solution before and after the addition of 4.5 eq. of different anions or together with F^−^ (lower, right corner). **(B)** Photographs of ABDF in various solutions before and after the addition of 4.5 eq. of F^−^. **(C)** Normalized UV/Vis spectra of ABDF in various organic solutions before and after addition of 4.5 eq. of F^−^. **(D)** Proposed mechanism for single fluoride anion detection of ABDF, and the partial ^1^H NMR titration spectra of ABDF in CDCl_3_ in the presence of 5 eq. of TBAF.

For the purposes of examining the sensing selectivity of ABDF to F^−^, a THF solution containing other anions, namely Cl^−^, Br^−^, I^−^, ${\text{NO}}_{3}^{-}$, ${\text{SO}}_{4}^{2-}$, SCN^−^, ${\text{ClO}}_{4}^{-}$, ACO^−^, and H_2_${\text{PO}}_{4}^{-}$ (as tetrabutylammonium salts), was used. Figure 2A shows that ABDF solution does not change color when any additional anions except for F^−^ are added. Mixing the solution with multiple anions afforded no noticeable color change. Once F^−^ is added into the mixture, the color changes immediately from blue to orange (Figure 2A). This indicates that ABDF is a highly selective sensor for F^−^ and that the presence of other anions does not alter its effectiveness.

Figures 2B,C show photographs and normalized UV/vis spectra of ABDF in various common organic solutions with different polarities. ABDF exhibits positive solvatochromic behavior and various colors in different solvents (Figure 2B). The UV/Vis spectra of ABDF exhibit a red-shift from non-polar solvent to polar solvent (Figure 2C), which could be ascribed to several specific and non-specific solute^−^solvent interactions. As can be seen from Figure S1, the proton signals of ABDF in DCM-d_2_ are shifted to the low field compared with in DMSO-d_6_. It should be noticed that the signals from amino (NH) units with a chemical shift of 4.34 ppm moved to 7.06 ppm. This could be ascribed to change in the chemical structure; a suggested mechanism is shown in Figure S2. Interestingly, the ABDF solution exhibited various colors (colorless, blue, orange, red) in different F^−^ solvents (Figure 2B). This could provide ABDF with detection performance not only for F^−^ but also for the polarity (polar or non-polar) of the solvent of F^−^. To the best of our knowledge, this is the first time that sensor detection of fluoride anions in different organic solvents, showing varied colors, has been reported.

In order to further investigate the detection performance of ABDF for F^−^ in polar solvent, we measured and analyzed a series of UV/Vis spectra of ABDF with a concentration of 1.0 × 10^−5^ M in DMSO in the presence of F^−^ from 0.0 to 4.5 eq. and the absorption intensity ratio of ABDF (5 × 10^−6^ M in DMSO) between 673 and 494 nm vs. F^−^ concentration (Figures S3, S4). Similar to Figure 1, with progressive addition of fluoride anions, the absorption intensity of the spectral peak at 673 nm gradually decreases and finally disappears, while the absorption intensity at 494 nm steadily increases. The inset of Figure S3 shows that the color of the ABDF solution is changed from dark blue to red upon adding F^−^. The color change is significantly different to that of ABDF in THF solution with F^−^. We assumed that the different colors originated from different chemical structures (Figure S2). Figure S4 shows that the detection limit of ABDF for F^−^ in DMSO (polar solvent) is as low as 1.0 × 10^−6^ M. ABDF is a sensitive fluoride anion sensor allowing naked-eye detection in various organic solvents. After F^−^ sensing, the UV/Vis absorption spectra exhibited a blue-shift (Figure 2C). To the best of our knowledge, most F^−^ sensors exhibit a red-shift with F^−^ detection (Lee et al., 2001; Han et al., 2007; Yang et al., 2013; Sun et al., 2017). Figure 2C shows that the UV/Vis absorption of ABDF exhibits a blue-shift over 200 nm once it has interacted with F^−^. This could be ascribed to the donor ability of the amino units being weaker after sensing F^−^, which decreases the push^−^pull electron effect within the ABDF molecular structure.

The color change of ABDF in the presence of F^−^ may be due to the deprotonation of the amino moiety (NH) by F^−^ (inter-molecular proton transfer, IPT; Figure 2D, Figure S2). To confirm our assumption and further understand the interaction between F^−^ and the donor, ^1^H NMR experiments were carried out in CDCl_3_. As shown in Figure 2D, the specific signal for amino protons at 7.06 ppm disappeared, and other proton signals from the core of ABDF shifted to lower ppm. This indicates the occurrence of an IPT event and the formation of hydrogen bonding between F^−^ and the proton on the amino N-H. ABDF is electronically neutral in the absence of F^−^, while a negatively charged species with one negative charge is formed in the presence of F^−^ (Figure 2D).

## Conclusion

In this article, a new kind of colorimetric chemosensor based on aminobenzodifuranone that has high selectivity and sensitivity for F^−^ was developed. ABDF responds to only F^−^ among various anions and has a detection limit as low as 5.0 × 10^−7^ M. A large absorption blue-shift of 200 nm occurred upon adding F^−^, which is manifested as a color change from dark blue to various colors (colorless, yellow, orange, and red) in different common organic solvents. All the colors are detectable by the naked eye under ambient light, which means the ABDF is not only a highly sensitive and selective F^−^ sensor allowing naked-eye detection, but it can also detect the polarity (polar or non-polar) of F^−^ solvents. The analyses of ^1^H NMR titration spectra have confirmed that F^−^ causes deprotonation of the amino moiety. ABDF is a promising colorimetric chemosensor for fluoride anion.

## Data Availability Statement

All datasets generated for this study are included in the article/Supplementary Material.

## Author Contributions

ZD and RL prepared materials and carried out in experiments. JG, MZ, and LL helped to analyze experimental data. XS, WR, ZM, and ZJ helped to characterize materials. JH supervised the work. All authors contributed to revise the manuscript, approved the final version and agreed to be accountable for all aspects of this work.

### Conflict of Interest

MZ was employed by company Qingdao Haiwan Science and Technology Industry Research Institute Co., Ltd. The remaining authors declare that the research was conducted in the absence of any commercial or financial relationships that could be construed as a potential conflict of interest.
