# Supplementary material for: Efficient Colorimetric Fluoride Anion Chemosensors With Varied Colors Based on Simple Aminobenzodifuranone Organic Π-Conjugated Dyes
Source: Front Chem. 2020 Apr 15;8:231. doi: 10.3389/fchem.2020.00231 (PMC7175790; doi:10.3389/fchem.2020.00231)
Supplement: Supplementary file 1 [file Data_Sheet_1.pdf]

Supporting Information

Efficient Colorimetric Fluoride Anion Chemosensors with Varied Colors Based-on Simple Aminobenzodifuranone Organic π-conjugated Dyes

Zhi Feng Deng [1a], Rui Li [2a], Jie Ting Geng [2], Meng Zheng,[3] Lei Quan Li [1], Xin Shi [1], Wen Qi Ren [1], Zi Yue Meng [1], Zhuo Ting Ji [1], Jing Hua [2*]*

[1] National and Local Joint Engineering Laboratory for Slag Comprehensive Utilization and Environmental Technology, School of Materials Science and Engineering, Shaanxi University of Technology (SNUT), Hanzhong 723000, PR China

[2] Key Laboratory of Rubber-Plastics of Ministry of Education/Shandong Province, School of Polymer Science & Engineering, Qingdao University of Science & Technology, Qingdao, Shandong 266042, PR China

[3] Qingdao Haiwan Science and Technology Industry Research Institute Co., Ltd., Qingdao, Shandong 266031, PR China

[a] Zhifeng Deng and Rui Li contributed equally to this work.

*Correspondence: Zhi Feng Deng dengzf@snut.edu.cn;
Jing Hua huajing72@qust.edu.cn;

Contents

1. Experimental Procedures

Materials.

Instrumentaion.

Sensitive for fluoride anion.

Scheme S1: Synthetic route to compound **a**.

Scheme S2: Synthetic route to compound **b**.

Scheme S3: Synthetic route to compound aminobenzodifuranone (ABDF).

2. Figures

Figure S1: ^{1}H NMR spectra of ABDF in DCM-d_2 and DMSO-d_6, respectively. **Figure S2:** Suggested mechanism of ABDF in different solvents.

Figure S3: The UV/vis absorption spectra of ABDF (1.0×10^{-5} M) in the presence of F^- (0–4.5 equiv.) in DMSO.

Figure S4: Absorption intensity ratio of ABDF (5×10^{-6} M in DMSO) between 673 nm

and 494 nm (A_{673} nm / A_{494} nm) versus fluoride anion concentration.

Materials

Reagents. 4-Bromo-mandelic acid, alloxan monohydrate, hydroquinone, *N*-ethylaniline, and potassium hydroxide were obtained from Aldrich and Fluka and used without further purification. Solvents were of analytical grade and used without further purification.

Instrumentation. UV/vis absorption spectra were recorded using a dual-beam grating PerkinElmer Lambda 950 UV/vis absorption spectrometer with a 1 cm quartz cell. ^{1}H NMR spectra were recorded using a Bruker DPX 500 spectrometer, which operates at 500 MHz.

Sensitive for fluoride anion:

Different concentration of fluoride anion in THF solvent (3 ml) was added to ABDF (1×10^{-5} M, THF, 3 ml). The concentration of ABDF in this mixture was 5.0×10^{-6} M, while the concentration of fluoride anion was reduced by half compare to the previously solvent. The UV/vis spectra of the mixture with 5.0×10^{-6} M ABDF and different concentration of fluoride anion was measured after the mixing two component for half hour. This experiments was also measured in the DMSO.

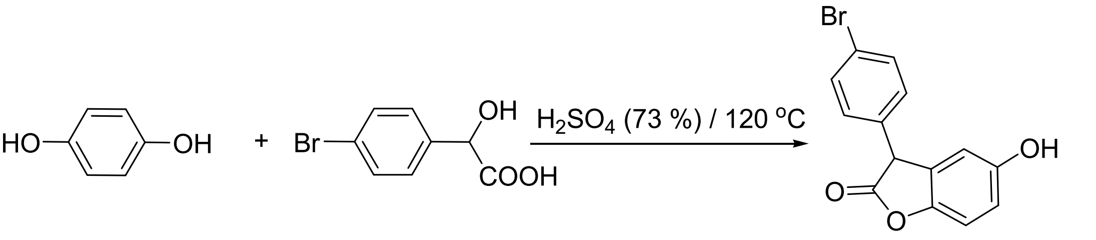

a

Scheme S1: Synthetic route to compound **a**.

5-Hydroxy-2-oxo-3-(4-bromophenyl)-2,3-dihydrobenzo[1,2-*b*]furan (a)

In a 50 ml flask, 4-bromo-mandelic acid (2.5 g, 11 mmol) and hydroquinone (3.58 g, 32 mmol) were stirred in 73 % sulphuric acid (10 ml) at 120 °C for 30 min. After cooling, the mixture was poured carefully into ice water (100 ml). The resulting white solid was filtered off and washed with water until acid free. The product was dried in air, recrystallized from toluene. A white powder product was obtained (2.9 g, 87 %). ^{1}H NMR (300 MHz, d$_6$-Aceton) δ ppm: 7.56–7.59 (d, 2H, J = 8.4 Hz), 7.22–7.25 (d, 2H, J = 8.4 Hz), 7.05–7.08 (d, 2H, J = 8.7 Hz), 6.64–6.69 (m, 1H), 6.72–6.74 (m, 1H), 5.16 (s, 1H). ^{13}C NMR (300 MHz, d$_6$-Acetone): 176.70, 156.31, 148.67, 137.43, 133.84, 132.22, 129.98, 123.31, 117.47, 113.97, 112.88, 51.24. Elemental microanalysis found C, 55.13; H, 2.98 % (C,55.11 %; H, 2.97 %).

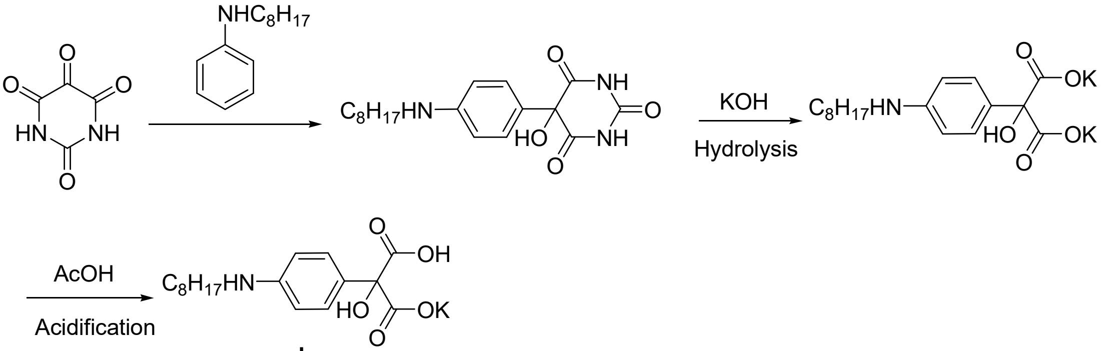

b

Scheme S2: Synthetic route to **b**.

4-(Octyl-amino)phenyltartronic acid (b)

Alloxan monohydrate (3.2 g, 20 mmol) and *N*-octyllaniline (4.1 g, 20 mmol) were dissolved in a mixture of 36 ml ethanol and 6 ml water. While stirring the clear yellowish solution was acidified with acetic acid (2 ml, 34 mmol). Stirring was continued at 60 ^{o}C for 5 hours. the solvent was removed upon vacuum evaporation after the reaction. The raw mixture was dissolved in a small amount of ethanol and precipitated in water. A fawn flaky substance was obtained after decantation and drying with a 48 % (3.02 g) yield of 5-hydroxy-5-(4octyllaminophenyl)-barbituric acid. The obtained material were then dissolved in an aqueous potassium hydroxide solution (5.5 g, 97.9 mmol, 1.4 M), and stirred between 40 and 45 ^{o}C for 5 hours. The solution was acidified with acetic acid and water was added. The product was stayed overnight to allow for precipitation of the product. After filtrated and dried, a light brown power product was obtained (1.03 g, 4.32 mmol, yield 45 %). ^{1}H NMR (500 MHz, d_6-Acetone) δ ppm: 7.04–7.07 (d, 2H, J = 8.4 Hz), 6.53–6.58 (d, 2H, J = 8.7 Hz), 6.28–6.29 (d, 1H, J = 7.7 Hz), 5.84 (s, 1H), 2.99 (s, 2H), 1.51–1.53 (t, 2H), 1.27 (s, 10H) 0.87 (t, 3H, J = 7.2 Hz). ^{13}C NMR (125 MHz, d_6-Acetone): 172.91, 154.90, 149.90, 128.33, 118.87, 112.71, 82.79, 43.70, 31.85, 29.40, 27.14, 22.69, 14.14. Elemental microanalysis found C,56.47 %; H, 6.72 %; N 3.84 % (C,56.49 %; H, 6.69 %; N, 3.87 %).

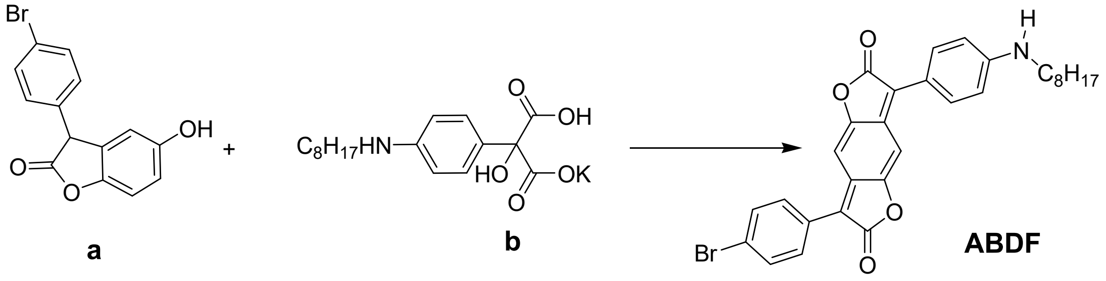

Scheme S3: Synthetic route to aminobenzodifuranone.

Aminobenzodifuranone (ABDF)

a (1.39 g, 4.6 mmol) and **b** (2.48 g, 7.2 mmol) were stirred in acetic acid (20 ml). The mixture was heated to 110 °C and stirred for 5 h at this temperature. 1.05 g (4.6 mol) of ammonium persulphate was added in 8 portions at 80 °C. The mixture was stirred at 95°C for 1 h. Subsequently, the mixture was cooled to room temperature and a small amount of water was added. The precipitate was filtered, washed with a small amount of acetic acid, and then with water. The crude product was refluxed methanol giving a dark solid (1.05 g, yield: 42%). ^{1}H NMR (500 MHz, $CDCl_3$) d ppm: 7.83–7.96 (d, 2H), 7.65–7.72 (m, 4H), 6.99 (s, 1H), 6.82 (s, 1H), 6.69–6.72 (d, 2H), 4.37 (s, 1H), 3.22–3.27 (t, 2H), 1.64–1.72 (m, 2H), 1.32 (s, 10H), 0.89–0.95 (t, 3H). Due to the moderate soluble of ABDF in DMSO, the ^{13}C NMR could be not measured. Elemental microanalysis found C,65.89 %; H, 5.22 %; N 2.58 % (C,65.94 %; H, 5.16 %; N, 2.56 %).

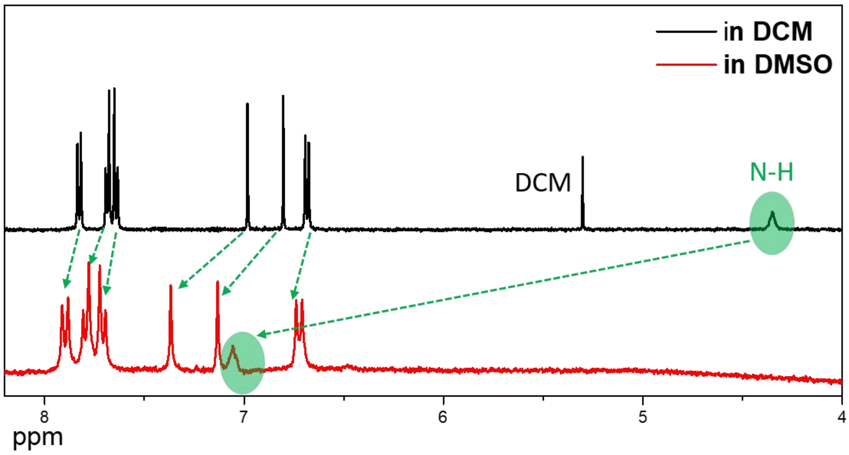

Figure S1: ^{1}H NMR spectra of ABDF in DCM-d_2 and DMSO-d_6, respectively.

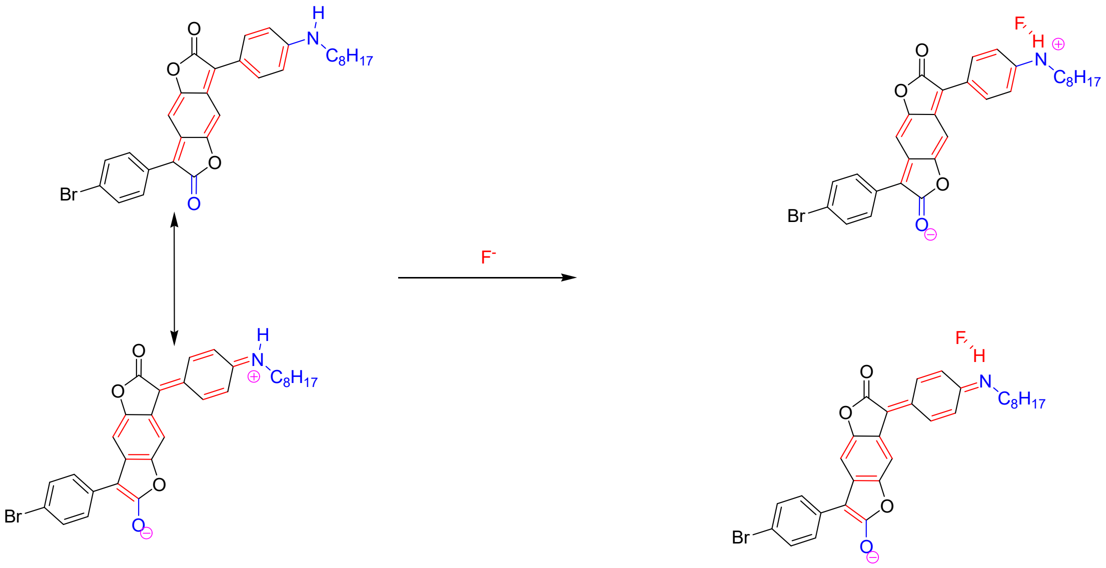

Figure S2: Suggested mechanism of ABDF in different solvents and fluoride anion sensing.

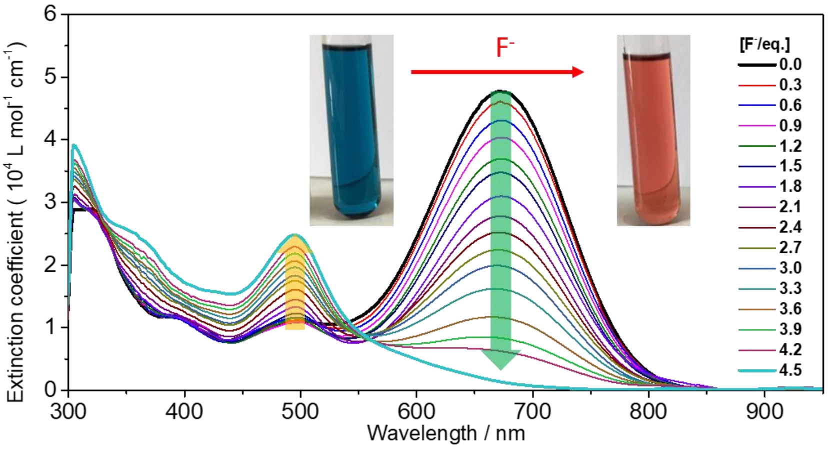

Figure S3: The UV/vis absorption spectra of ABDF (1.0×10^{-5} M) in the presence of F^- (0–4.5 equiv.) in DMSO. With progressive addition of fluoride anion added, the absorption intensity at 673 nm was gradually decreased and finally this peak disappeared, while the absorption peak at 494nm was steadily increased. The inset shows the color change from dark blue to red6 of ABDF upon adding F^- (left: free ligand; right: 4.5 eq. F^-).

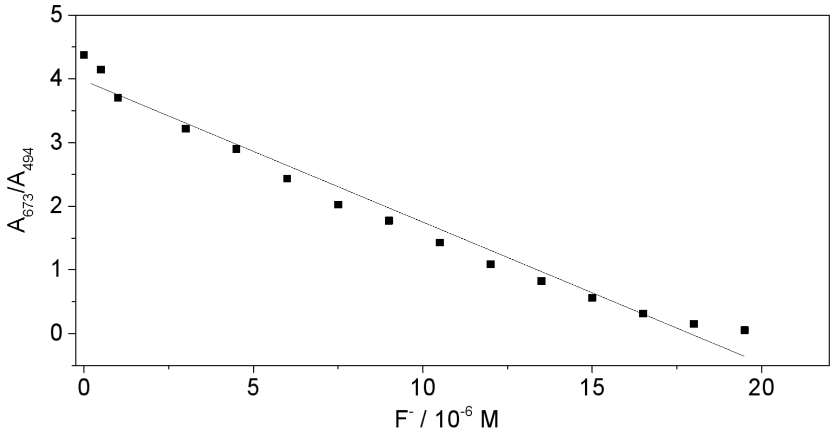

Figure S4: Absorption intensity ratio of ABDF (5×10^{-6} M in DMSO) between 673 nm and 494 nm (A_{673} nm / A_{494} nm) versus fluoride anion concentration. there is a slope correlation. The signal output discrimination was still clear at F^- concentration at 1.0 $\times$ 10^{-6} M, which indicates that the sensor was highly sensitive to F^- concentration change and could offer a quantitative information in DMSO.
